# Supplementary material for: ProtMamba: a homology-aware but alignment-free protein state space model
Source: Bioinformatics. 2025 Jun 13;41(6):btaf348. doi: 10.1093/bioinformatics/btaf348 (PMC12206526; doi:10.1093/bioinformatics/btaf348)
Supplement: btaf348_Supplementary_Data [file btaf348_supplementary_data.pdf]

Supplementary material for "ProtMamba: a homology-aware but alignment-free protein state space model"

Damiano Sgarbossa[†], Cyril Malbranke[†], Anne-Florence Bitbol[*]

Institute of Bioengineering, School of Life Sciences, École Polytechnique Fédérale de Lausanne (EPFL), Lausanne, Switzerland
SIB Swiss Institute of Bioinformatics, Lausanne, Switzerland
[†]These authors contributed equally to this work.
[*]Corresponding author: anne-florence.bitbol@epfl.ch

June 3, 2025

A ProteinGym assays used in validation

Here, we list the 20 assays we extracted from the ProteinGym benchmark to choose some hyperparameters (see Figure S11).

A0A2Z5U3Z0_9INFA_Wu_2014
AMFR_HUMAN_Tsuboyama_2023_4G3O
CAR11_HUMAN_Meitlis_2020_lof
CBS_HUMAN_Sun_2020
CUE1_YEAST_Tsuboyama_2023_2MYX
DYR_ECOLI_Nguyen_2023
GDIA_HUMAN_Silverstein_2021
HIS7_YEAST_Pokusaeva_2019
HXK4_HUMAN_Gersing_2023_abundance
KCNE1_HUMAN_Muhammad_2023_expr
KKA2_KLEPN_Melnikov_2014
PITX2_HUMAN_Tsuboyama_2023_2L7M
PPM1D_HUMAN_Miller_2022
R1AB_SARS2_Flynn_2022
RDRP_I33A0_Li_2023
S22A1_HUMAN_Yee_2023_abundance
SCN5A_HUMAN_Glazer_2019
SHOC2_HUMAN_Kwon_2022
TRPC_SACS2_Chan_2017
VILI_CHICK_Tsuboyama_2023_1YU5

B Details on mutational effect prediction with ProtMamba

In Table 2 of the main text, we consider different ways of computing mutational effects using ProtMamba, and compare with other models. Here, we explain our different approaches in more detail.

- The entry **ProtMamba (single)** of Table 2 is the result reported when using the FIM technique at the end of the sequence to evaluate mutations. It is the fastest method among homology-aware ones. The procedure is the following:
  1. Subsample a predetermined number of homologs of the target sequence considered in the DMS to be used as context, based on a diversity filter.
  2. Run ProtMamba on the context and collect the last hidden state of the context (as the model is recurrent).
  3. Start from the last hidden state as initial state for every variant to score. Scoring is then very fast, since we only need to apply ProtMamba on a single sequence per variant, and not on the full context. As Mamba scales linearly in sequence length, this allow to evaluate many different variants very fast on a single GPU (hundreds to thousands per batch). The mutated residues are put at the end of the sequence in the FIM mask. For single mutations, we can then evaluate in one shot the likelihood of every mutation.
  4. We compare the likelihood of the WT to the likelihood of the variant using Fill-in-the-middle to get a proxy of variant fitness. We can then evaluate the fitness of variants comprising each of the 20 amino-acids at the mutated site(s) in a single shot.
- The entry **ProtMamba AR (autoregressive)** of Table 2 is the result reported when evaluating the likelihood of a variant without using the FIM technique. The procedure is:
  1. Perform steps 1 and 2 as in ProtMamba (single) above.

2. Start from the last hidden state as initial state for every variant to score. We then evaluate the autoregressive likelihood of the full variant. Since some logits are computed after the mutation in this setup (because they are positioned after in the sequence), we cannot evaluate the fitness of the 20 amino-acids in a single shot (in contrast to the previous approach), which increases the number of calls to ProtMamba, and hence requires more time.
3. Compare the likelihood of the WT over the full sequence to the likelihood of the variant to get an evaluation of variant fitness.

- The entry **ProtMamba (w/ R), i.e. with retrieval** of Table 2 is the result reported when combining ProtMamba with a prior based on the frequency of amino-acids in the MSA of the relevant protein family.
  1. Perform steps 1, 2 and 3 as in ProtMamba (single) above.
  2. Load the MSA, compute a log-likelihood prior ($\log p_{\text{retrieval}}$) based on the frequency of every amino-acid, and sum it with ProtMamba's log-likelihood ($\log p_{\text{ProtMamba}}$) :

$$\log p_{\text{ProtMamba (w/R)}} = \alpha \log p_{\text{retrieval}} + (1 - \alpha) \log p_{\text{ProtMamba}} .$$

  The parameter α was optimized using the validation set introduced in Section A (see Figure S11). This operation can be parallelized across CPUs or GPUs. We report the results using 16 workers.

Note that we tested ProtMamba AR both with our model fine-tuned on FIM (ProtMamba Long Finetuned) and with our foundation model (ProtMamba Long), and we obtained similar results (respectively 0.361 and 0.367).

In Table S1, we break down results by MSA depth and by number of mutations. We observe that for datasets with more than one mutation (last column in Table S1), ProtMamba with retrieval slightly outperforms the overall state-of-the-art model TranceptEVE L and reaches performance close to the structure-based model ESM-IF1. However, averaging over all datasets, ProtMamba does not reach the same performance as TranceptEVE L. But since ProtMamba performs better than Tranception L, ensembling ProtMamba and EVE predictions might yield comparable performance.

		Spearman correlation by MSA depth				by mutations	
**Model**	**Par.**	**All depths**	**Deep**	**Medium**	**Shallow**	**1**	**2+**
ESM-2	150M	0.387	0.497	0.358	0.306	0.367	0.379
ESM-IF1	142M	0.422	0.544	0.431	0.300	0.413	0.471
Tranception S (w/o R)	85M	0.303	0.320	0.295	0.258	0.293	0.262
Tranception L (w/o R)	700M	0.374	0.419	0.371	0.358	0.358	0.390
**ProtMamba (w/o R)**	107M	0.406	0.465	0.411	0.391	0.376	0.444
MSA Transformer	100M	0.421	0.473	0.435	0.393	0.392	0.435
Tranception S (w/ R)	85M	0.418	0.444	0.415	0.428	0.389	0.409
Tranception L (w/ R)	700M	0.434	0.473	0.438	0.432	0.404	0.463
TranceptEVE L	>700M	0.456	0.492	0.467	0.451	0.426	0.467
**ProtMamba (w/ R)**	107M	0.432	0.472	0.438	0.448	0.404	0.469

Table S1. **Performance of different models on the ProteinGym benchmark.** We report Spearman correlation values obtained both based on retrieval (w/ R) and non-retrieval (w/o R) methods, and parameter count for each model. We report results divided according to MSA depth and number of mutations in the benchmark dataset. Results for benchmark models were obtained from `https://proteingym.org/`. Note that PoET-205M [1] reports an overall Spearman correlation of 0.474 [2] on ProteinGym, but it is not yet on the ProteinGym website, and no information is given about the training time or resources.

C Ablation Studies

We investigated ablations or alternative implementations of ProtMamba using two models: a small model with 14 million parameters (8 layers, hidden dimension 512) and the standard architecture with 107 million parameters (16 layers, hidden dimension 1024). Both models were trained for 10 billion tokens (50k steps with a batch size of 128) and evaluated on a validation set of 500 unseen clusters. During evaluation, a context of 25 sequences was used. The perplexity of the models was assessed both autoregressively (left-to-right) and in the fill-in-the-middle (FIM) spans.

The performance of these alternatives (described in more detail below) is summarized in Table S2. Perplexity values are reported for both autoregressive and FIM modes in the small model and the larger one.

Perplexity	14M Parameters		107M Parameters	
	Autoregressive	FIM	Autoregressive	FIM
Only FIM from scratch	Fail	13.90 ± 0.34	Fail	15.59 ± 0.27
AR only	**12.58** ± 0.31	18.03 ± 0.25	11.05 ± 0.36	Fail
No positional encoding	13.01 ± 0.30	16.71 ± 0.47	12.31 ± 0.37	17.20 ± 0.58
Additive positional encoding	12.72 ± 0.31	13.60 ± 0.33	12.58 ± 0.38	13.81 ± 0.31
One mask, one token	12.76 ± 0.31	15.54 ± 0.29	11.04 ± 0.33	16.60 ± 0.36
Masking fraction 50%	13.02 ± 0.31	**13.44** ± 0.33	**10.94** ± 0.36	**11.59** ± 0.35
ProtMamba	13.00 ± 0.30	13.89 ± 0.32	11.35 ± 0.33	12.62 ± 0.30

Table S2. **Alternatives to ProtMamba.** Perplexity values are reported for different alternatives to ProtMamba, evaluated on small (14M parameters) and larger (107M parameters) models, for both autoregressive and FIM tasks.

The alternative implementations tested against our main ProtMamba model, and whose performance is reported in Table S2, were constructed as follows:

- **Only FIM from scratch:** This approach backpropagates the loss exclusively from the FIM tokens, disregarding the main amino-acid chain. Training this way from scratch disables autoregressive (left-to-right) next-token prediction and degrades performance, including on FIM tasks.
- **Autoregressive (AR) only:** Trains the model without sampling FIM spans. While this slightly improves autoregressive performance, it significantly degrades FIM capabilities.
- **No positional encoding:** Omits positional encodings entirely. In autoregressive mode, the model can partially rely on its recurrent architecture, but in FIM mode, performance suffers due to the absence of positional information in input.
- **Additive positional encoding:** Uses additive positional encoding (summing token embeddings with positional encoding) instead of concatenated positional encoding (concatenating token embeddings with positional encoding). This approach showed mixed results, with slight improvement in the small model but degradation in the larger model.
- **One mask, one token:** Uses one mask per token (as in the T5 model [3]) instead of one mask per span of tokens (as in our approach, inspired by [4]). This approach led to performance degradation in FIM, likely due to insufficient training on larger number of mask tokens.
- **Masking fraction 50%:** Samples 50% of the tokens for FIM (compared to ProtMamba's 20%). This alternative brought minor but noticeable improvements, suggesting potential for further development.

D Alternative Models

We compared Mamba against Hyena and GPT using flash attention, with small scale models. Models were built to have 14M parameters: 8 layers, 512 dimension representations (384 for GPT). We trained these models with context size 32.000 for 72h on single H100 GPUs, and we tested their perplexity on a held out validation set using both 16,000 and 120,000 context length. We report the results in Table S3.

Model	Training Time	Tokens Seen	Context Short		Context Long	
			PPL	Eval Rate	PPL	Eval Rate
Hyena (14M)	72h	81.9B	14.42	$16.6s^{-1}$	Fail	Fail
Mamba (14M)	72h	76.8B	12.48	$16.6s^{-1}$	11.89	$3.84s^{-1}$
GPT2 (14M)	72h	33.3B	10.68	$1.25s^{-1}$	11.72	$0.03s^{-1}$
ProtMamba (107M)	400h	190B	9.69	$8.33s^{-1}$	9.26	1.04

Table S3. Performance Metrics for Various Models, after 72h of training. Perplexity (PPL) and evaluation rate are reported using various architectures, for short (16,000 tokens) and long (120,000 tokens) context lengths.

E Supplementary figures

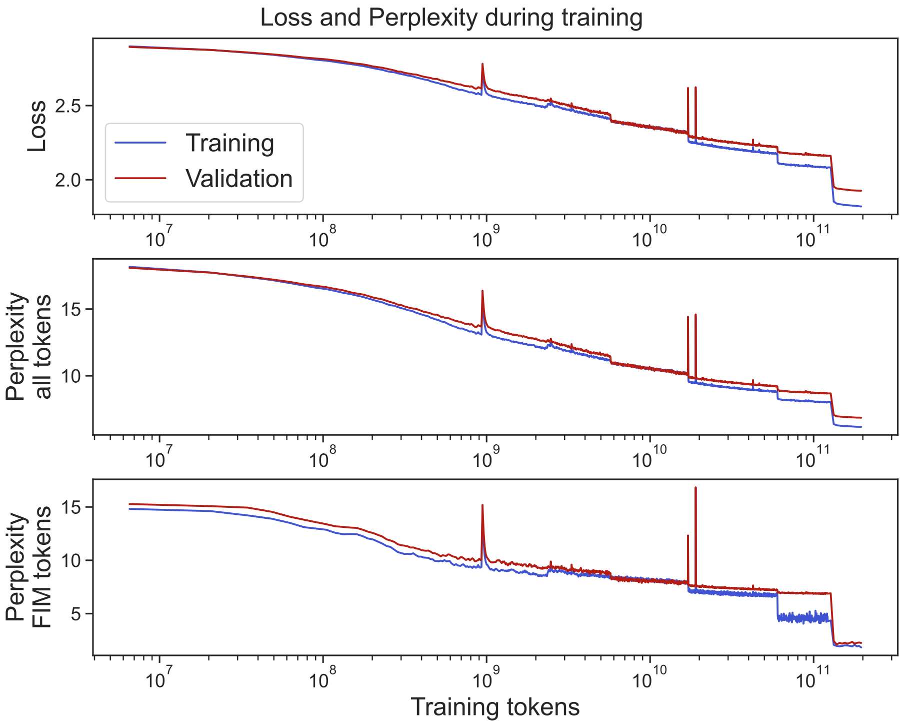

Fig S1. **Loss and perplexity during training.** Cross entropy loss and perplexity computed for both the full non-masked sequences and the FIM tokens. We show them as a function of the number of tokens processed during the training of ProtMamba. They are computed on the training set and on a validation set of 192 held-out OpenProteinSet sequence clusters (see Section 2.3 in the main text).

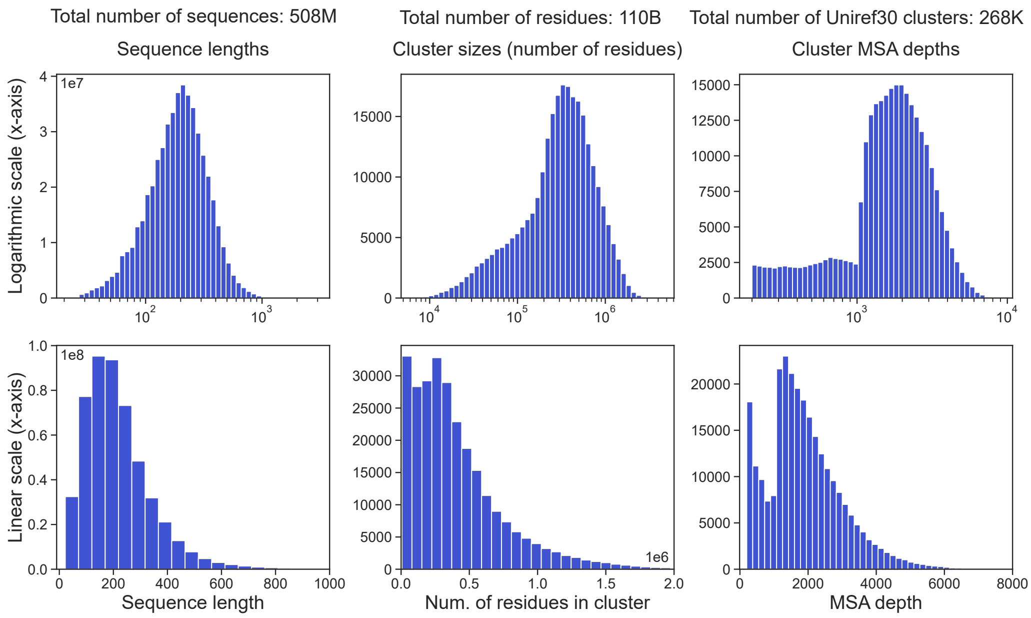

Fig S2. **Datasets statistics.** We show the x axis both in log scale (first row) and in linear scale (second row) to have a better grasp of the distributions.

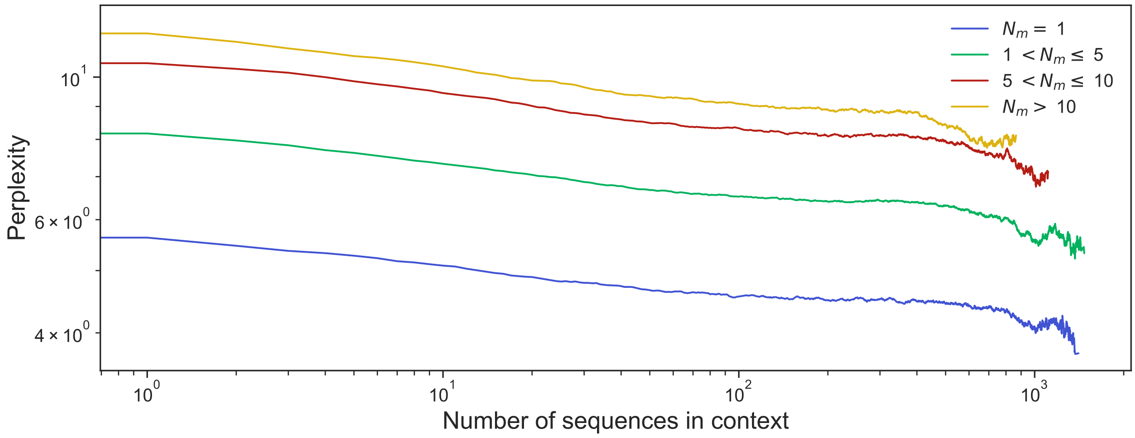

Fig S3. **Scaling of the FIM perplexity with the number of context sequences.** Same as Figure 2 in the main text, using logarithmic scales on both axes.

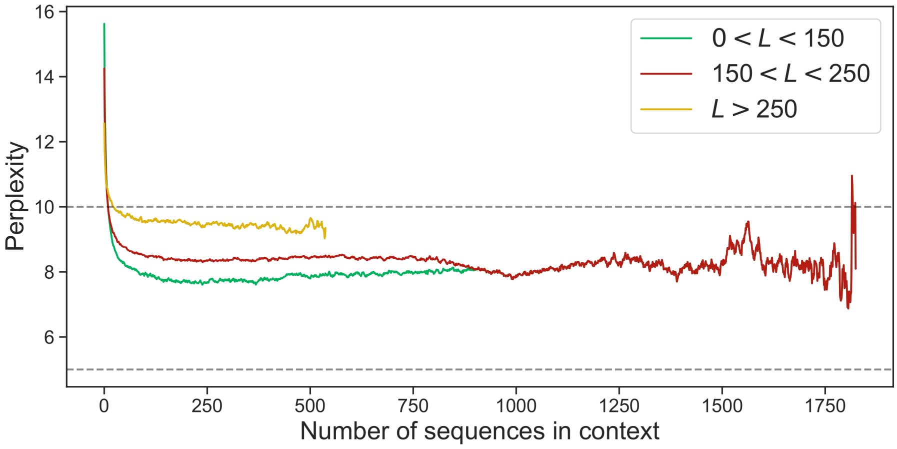

Fig S4. **Loss and perplexity of the full sequences vs. number of sequences in the context.** Scaling of the per-sequence perplexity (i.e. the standard autoregressive perplexity of the full non-masked sequence) versus the number of context sequences. Results are averaged over all 500 clusters of the test set and 20 replicates for each cluster (differing by the random sampling of context sequences). Context sizes go up to 2^{17} amino acids. Sequence clusters are split according to the average length L of sequences in the cluster. We observe that clusters with shorter sequences reach lower perplexities.

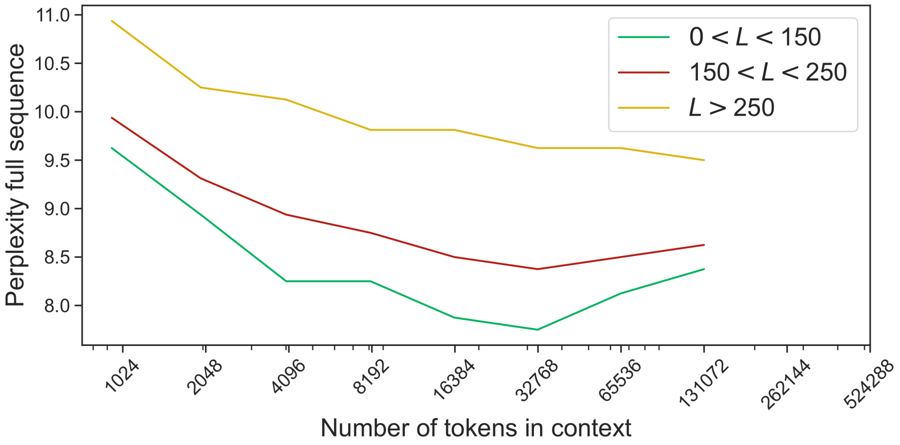

Fig S5. **Loss and perplexity of the full sequences vs. number of tokens in the context.** Scaling of the per-sequence perplexity (i.e. the standard autoregressive perplexity of the full non-masked sequence) versus the size of the context (i.e. the number of preceeding tokens). Results are averaged over all 500 clusters of the test set and 20 replicates for each cluster (differing by the random sampling of context sequences). Context sizes go up to 2^{17} amino acids. Sequence clusters are split according to the average length L of sequences in the cluster. We observe that clusters with shorter sequences reach lower perplexities.

Cluster	Hamming	HMMER	pLDDT	pTM
A0A2H9MP70	0.45	−0.44	−0.77	−0.54
A0A135YUE9	0.76	−0.68	−0.61	−0.58
G4ZH78	0.23	−0.42	−0.47	−0.47
A0A1A8YWK1	0.79	−0.7	−0.84	−0.81
A0A0A0HZM8	0.46	−0.47	−0.69	−0.63
A0A091TDH7	0.31	−0.32	−0.46	−0.46
A0A2N1P554	0.18	−0.35	−0.37	−0.4
A0A1C5UJ41	0.81	−0.78	−0.81	−0.77
A0A194V424	0.66	−0.71	−0.7	−0.54
S7UZ45	0.56	−0.57	−0.74	−0.66
F2CV06	0.89	−0.85	−0.81	−0.79
A0A146ZGL6	0.72	−0.75	−0.56	−0.64
D8SD16	0.65	−0.74	−0.71	−0.61
A0A139IN77	0.18	−0.2	−0.15	−0.27
A0A1C6Q5J2	0.55	−0.22	−0.45	−0.27
I4B642	0.44	−0.52	−0.61	−0.52
A0A2X4BAY2	0.27	−0.63	−0.63	−0.54
A0A241VGM5	0.44	−0.59	−0.62	−0.55
A0A1S3G530	0.88	−0.72	−0.8	−0.74
**Mean**	0.54	−0.56	−0.62	−0.57

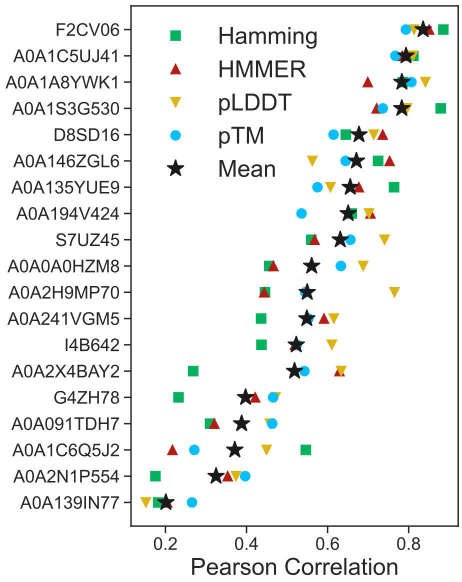

Fig S6. **Pearson correlation between ProtMamba perplexity and scores for generated sequences.** For each of 19 test clusters, we used all the sequences generated by ProtMamba to compute the Pearson correlation between the model perplexity and the Hamming distance to the closest natural neighbor, the HMMER score, the pLDDT and pTM scores from ESMFold.

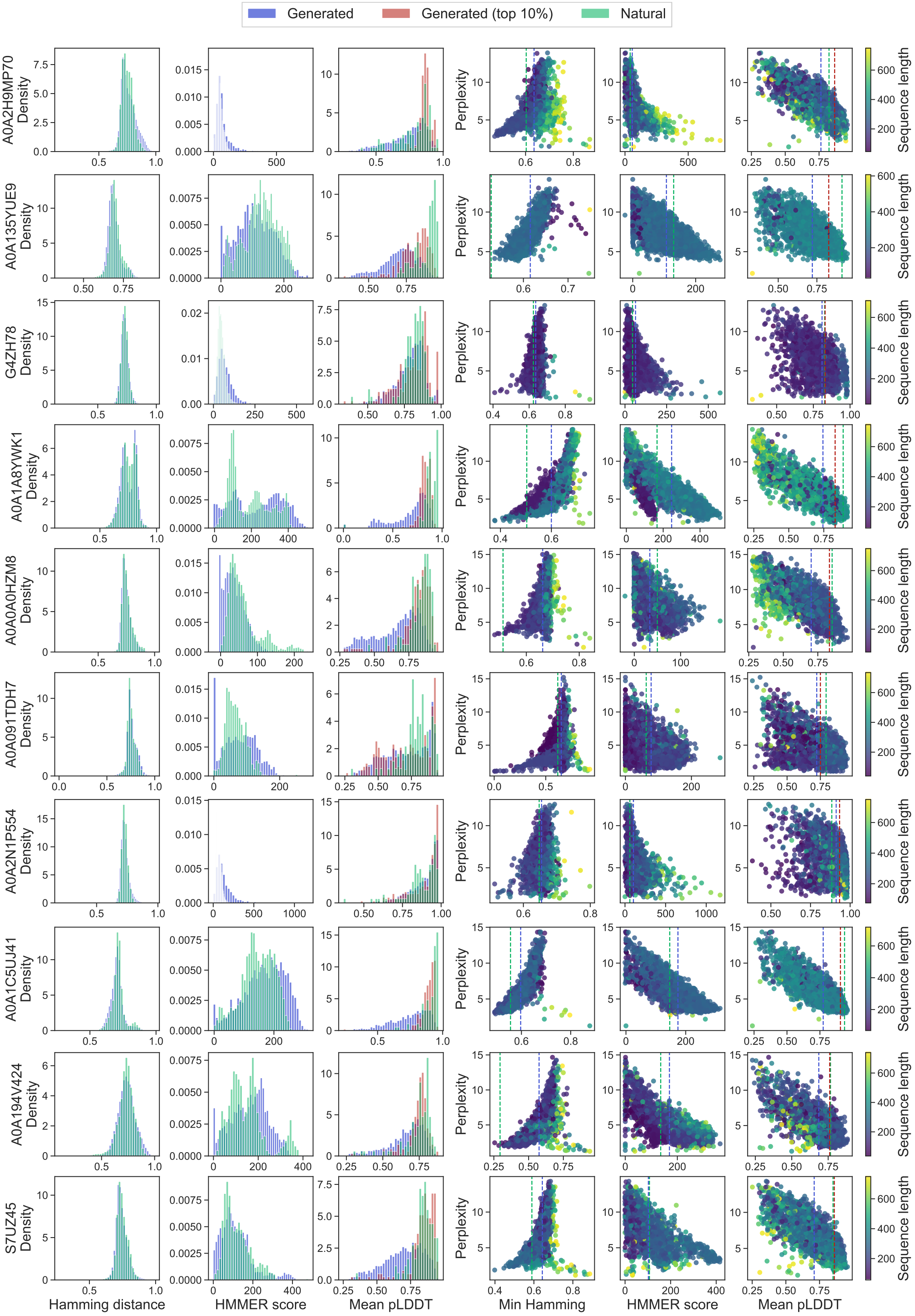

Fig S7. **Properties of generated sequences.** Left panels: histograms of Hamming distances, HMMER scores and mean pLDDT scores from ESMFold of generated sequences for 10 example test clusters (10 rows). Right panels: scatter plots of ProtMamba perplexity versus the Hamming distance to the closest natural neighbor, the HMMER score and mean pLDDT score from ESMFold for all generated sequences from each of 10 example clusters (10 rows). Dashed vertical lines: median of the generated sequences (blue), median of the natural sequences (green) and pLDDT value of the reference structure of the cluster (red). The last one is shown only for the rightmost plot.

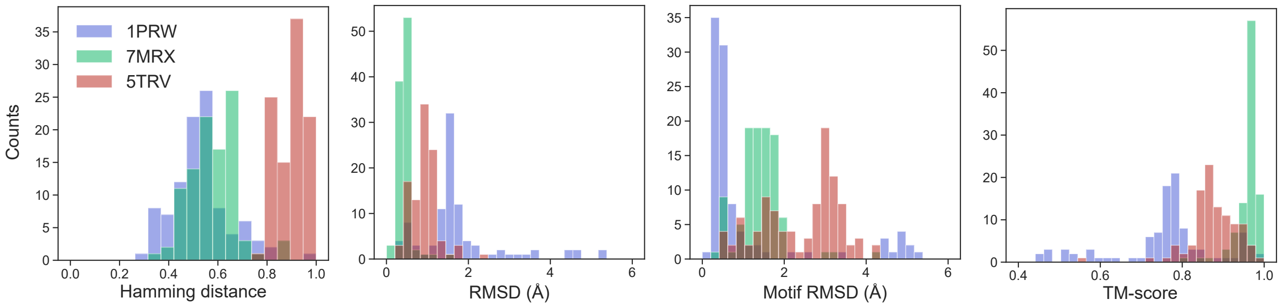

Fig S8. **Properties of inpainted functional motifs generated by ProtMamba.** For each of three protein structures (same as in Figure 4), we show the distribution of four scores over 100 inpainted sequences, where the masked functional motif was generated by ProtMamba. The four scores considered are: Hamming distance, illustrating sequence divergence; RMSD between the ESMFold-predicted structure of the inpainted sequence and the original experimental structure, calculated over the whole protein; RMSD calculated over the inpainted motif; TM-score. Since the protein family associated to 1PRW includes multiple conformations (e.g. 1CLL, 1A29, 1CKK), we computed TM-score and RMSD against the two most common conformations (1PRW and 1CLL), and reported respectively the highest and lowest values. Over 90% of the generated sequences achieved a TM-score ¿ 0.7 with at least one known conformation, indicating that ProtMamba reliably generates structurally plausible motifs even in the presence of different conformations.

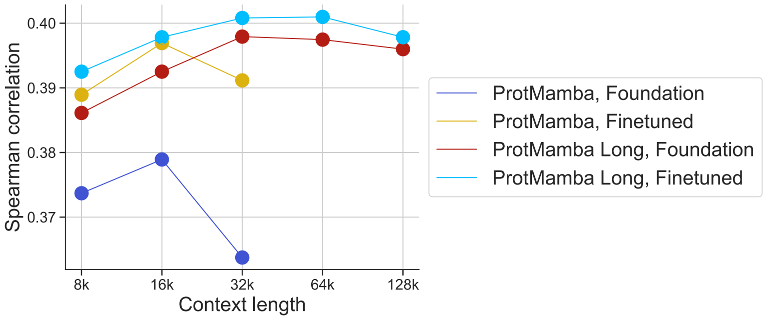

Fig S9. **Comparison of 4 ProtMamba variants on the ProteinGym benchmark.** We show the predictive power for variant effect on the ProteinGym benchmark, via the Spearman correlation between predictions and experimental results, for "ProtMamba, Foundation" ($2^{15} = 32768$ tokens context seen in training), "ProtMamba, Fine-tuned" (fine-tuned on predicting only FIM tokens), ProtMamba Long, Foundation" ($2^{17} = 131072$ tokens context seen in second phase of training) and "ProtMamba Long, Fine-tuned" (fine-tuned on predicting only FIM tokens). We notice that models fine-tuned only on the FIM objective outperform the foundation models. ProtMamba Long is overall performing better than ProtMamba and its performance does not decrease as sharply as ProtMamba for longer context.

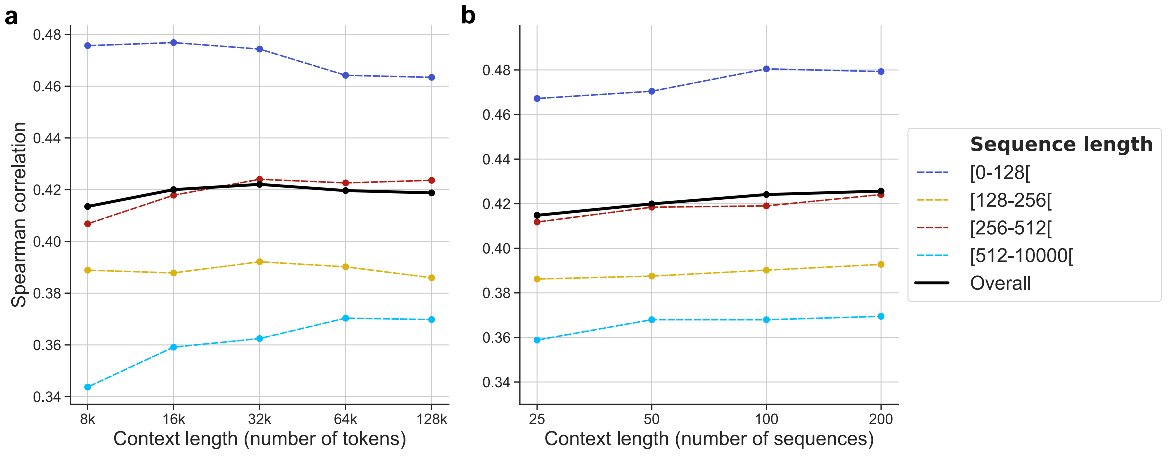

Fig S10. **Impact of context length on results on the ProteinGym benchmark.** **(a)** We run ProtMamba Long on the ProteinGym dataset, building contexts of different sizes in terms of numbers of tokens (from 8,000 to 128,000). We see that the increase in performance is more important for long sequences, which highlights the benefit of long context to model long protein sequences. **(b)** We also run ProtMamba Long on the ProteinGym dataset, building contexts of different sizes in terms of numbers of sequences (from 25 to 200). Overall, we notice a rise in the Spearman correlation, showing that prediction benefits from longer context.

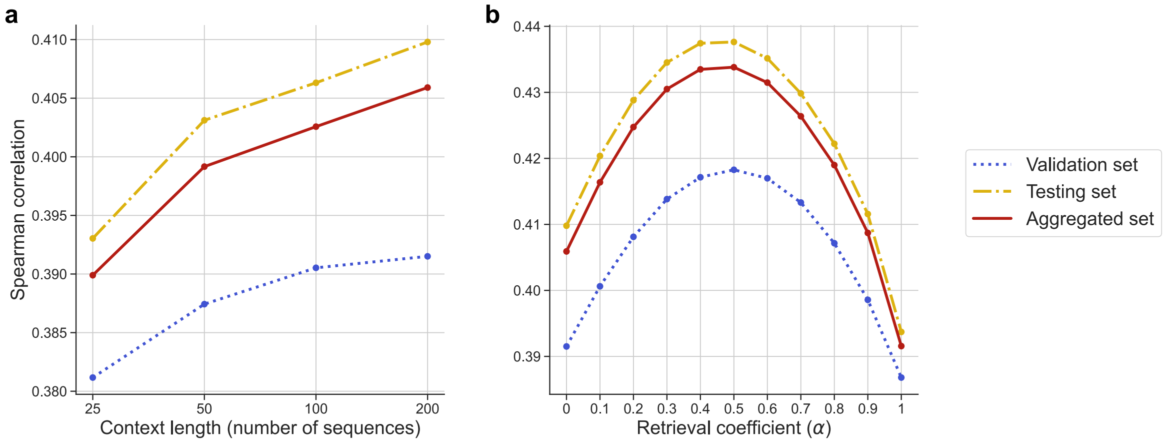

Fig S11. **Choice of context length and retrieval coefficient using a validation set.** We randomly extracted a validation set of 20 datasets (see supplementary Section A) to select the best context length and retrieval coefficient. **(a)** The prediction improves with the context size in the validation set. This trend was later observed in the rest of the benchmark (testing set) too. **(b)** Retrieval requires mixing the fitness score $\mathcal{F}_m$ obtained from ProtMamba and the fitness score obtained from the independent-site model $\mathcal{F}_i$ through the retrieval fitness score $\mathcal{F}_r = \alpha \mathcal{F}_i + (1 - \alpha)\mathcal{F}_m$. The best model on the validation set was obtained for a retrieval coefficient $\alpha = 0.5$, which was later verified on the rest of the dataset.

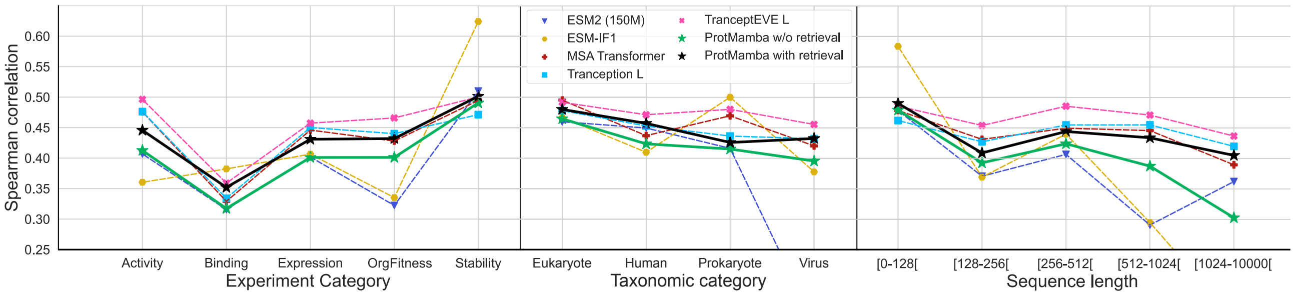

Fig S12. **Breakdown by categories of results on the ProteinGym benchmark.** Results of ProtMamba Long and of existing specialized models on the ProteinGym benchmark, averaged over all datasets, are shown broken down by category of experiments (left), taxonomic category (middle) and wild-type sequence length (right). ProtMamba is fairly competitive with these models. We note that the inverse folding model ESM-IF1 outperforms sequence-based models for stability assessment, as expected (see left panel).

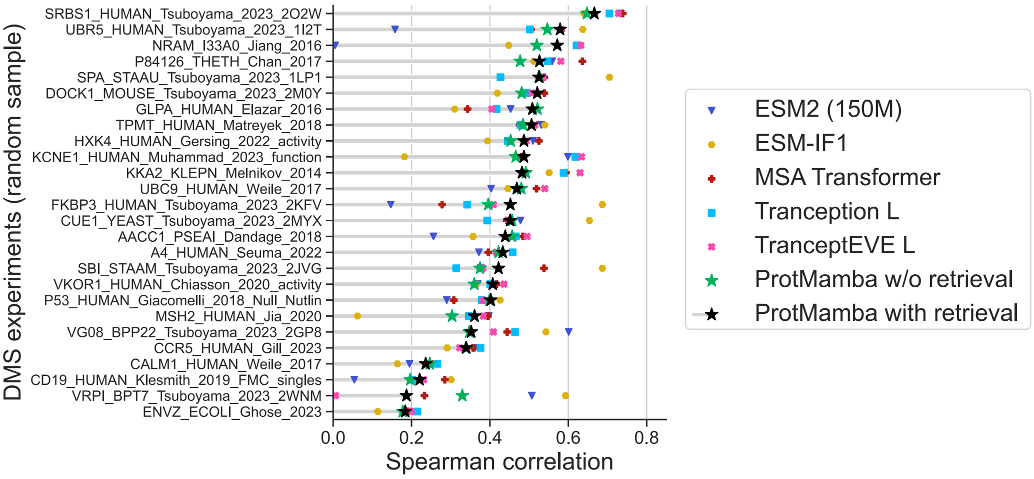

Fig S13. **Example results on the ProteinGym benchmark.** Results of ProtMamba Long are shown on 25 randomly sampled deep mutational scan (DMS) experimental datasets from ProteinGym, and are compared to existing methods (see main text). The score shown is the Spearman correlation between predictions and experimental results.

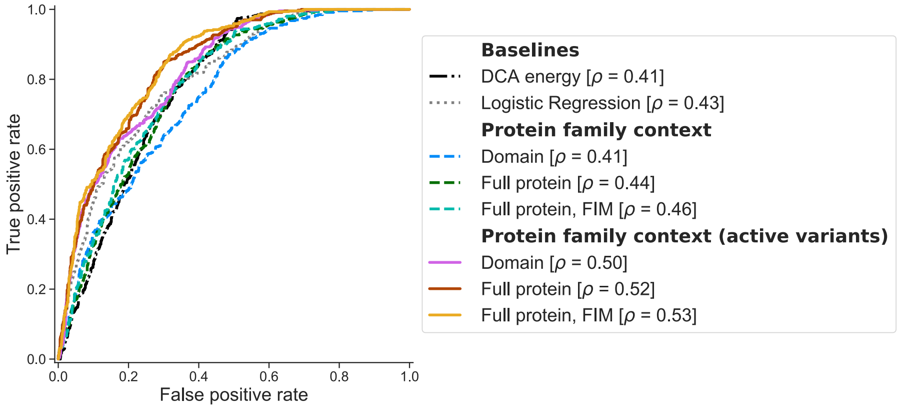

Fig S14. **Impact of various context construction methods on results on chorismate mutase activity.** The ROC curve is shown for various context construction methods (see main text) for predicting active variants in the chorismate mutase dataset, and for baseline methods from [5]. Overall, we observe that restricting to active variants in context helps improving prediction quality (Spearman correlation ρ going from 0.41-0.46 to 0.50-0.53). Giving full proteins instead of restricting to the chorismate mutase domain also improves the results. Using FIM to condition the domain to score using the rest of the protein also improves performance. ProtMamba also outperforms the baselines provided in [5], namely the Potts or DCA energy and the logistic regression trained directly on amino-acid sequences.

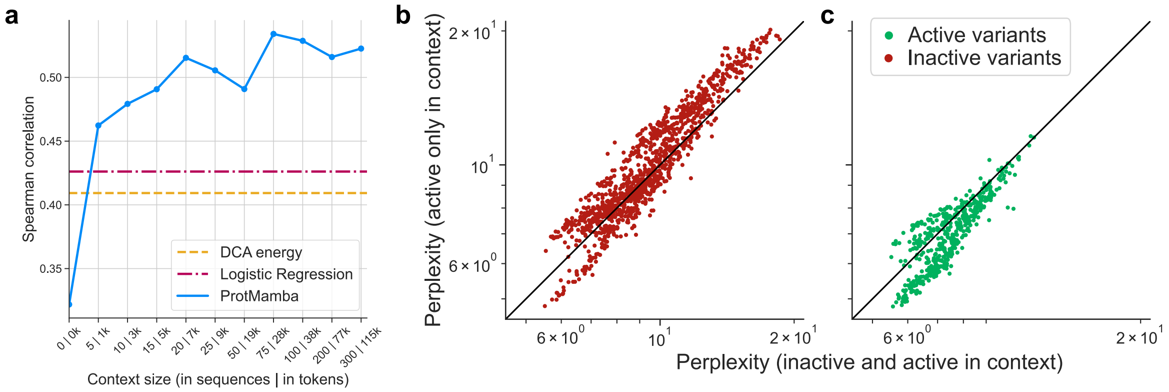

Fig S15. (**a**) **Impact of context length on results on chorismate mutase activity.** Spearman correlation between experimental activity and predictions from ProtMamba is shown using a different number of active sequences in the context (using FIM and full active proteins as context to score variants using ProtMamba). The Spearman correlation quickly increases with the number of proteins sequences given in context, especially from 0 to 25 sequences (or 10,000 tokens) before slowly increasing with context size. (**b**) and (**c**) **Perplexity of generated variants when using only active variants in context (b) or using active and inactive variants in context (c)**. Inactive variants tend to have higher perplexity (implying lower fitness score) when the context contains only active variants (**b**) while active variants have lower perplexity (implying higher fitness score) when the context contains only active variants (**c**) .

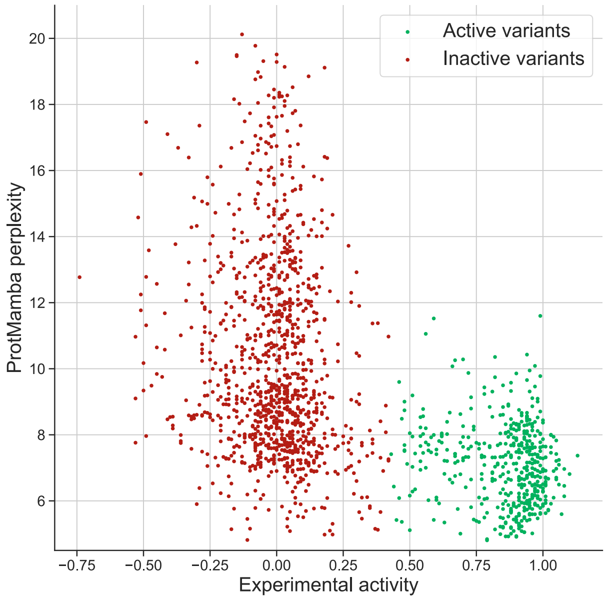

Fig S16. **ProtMamba captures chorismate mutase activity.** Experimental activity of chorismate mutase enzyme variants from [5] is shown versus ProtMamba per-token perplexity, determined using FIM and full active proteins as context. The per-token perplexity is a good proxy of the activity. We obtain a Spearman correlation of 0.53 between this score and experimental activity, and it yields an AUC of 0.84 to discriminate active from inactive sequences.

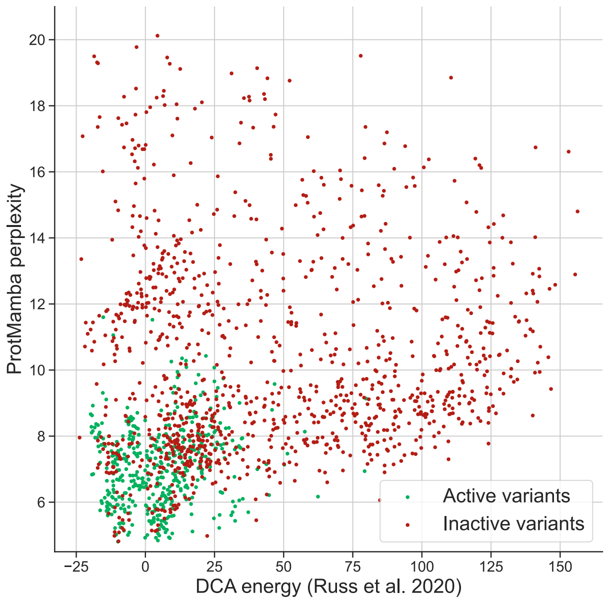

Fig S17. **ProtMamba perplexity versus DCA energy for chorismate mutase variants.** ProtMamba perplexity is evaluated using full sequences, FIM and only active variants in the context, and is shown versus the Potts or DCA energy from [5]. Active variants are in green, while inactive variants are in red. We observe that most of the variants that are active have low perplexity, and that many inactive variants that were not discriminated as inactive by DCA are labelled as such by ProtMamba (bottom right part of the plot).

References

1. Timothy Truong Jr and Tristan Bepler. PoET: A generative model of protein families as sequences-of-sequences. *Advances in Neural Information Processing Systems*, 36, 2024.

2. Timothy Truong Jr and Tristan Bepler. PoET: A high-performing protein language model for zero-shot prediction. https://www.openprotein.ai/poet-a-high-performing-protein-language-model-for-zero-shot-prediction.

3. Colin Raffel, Noam Shazeer, Adam Roberts, Katherine Lee, Sharan Narang, Michael Matena, Yanqi Zhou, Wei Li, and Peter J. Liu. Exploring the limits of transfer learning with a unified text-to-text transformer. *arXiv*, page 1910.10683, 2020.

4. Mohammad Bavarian, Heewoo Jun, Nikolas Tezak, John Schulman, Christine McLeavey, Jerry Tworek, and Mark Chen. Efficient training of language models to fill in the middle. *arXiv*, page 2207.14255, 2022.

5. William P. Russ, Matteo Figliuzzi, Christian Stocker, Pierre Barrat-Charlaix, Michael Socolich, Peter Kast, Donald Hilvert, Remi Monasson, Simona Cocco, Martin Weigt, and Rama Ranganathan. An evolution-based model for designing chorismate mutase enzymes. *Science*, 369(6502):440–445, 2020.
